# The aluminium content of infant formulas remains too high

**DOI:** 10.1186/1471-2431-13-162

**Published:** 2013-10-08

**Authors:** Nancy Chuchu, Bhavini Patel, Blaise Sebastian, Christopher Exley

**Affiliations:** 1 Life Sciences, Huxley Building, Keele University, Staffordshire, UK; 2 The Birchall Centre, Lennard-Jones Laboratories, Keele University, Staffordshire, UK

## Abstract

**Background:**

Recent research published in this journal highlighted the issue of the high content of aluminium in infant formulas. The expectation was that the findings would serve as a catalyst for manufacturers to address a significant problem of these, often necessary, components of infant nutrition. It is critically important that parents and other users have confidence in the safety of infant formulas and that they have reliable information to use in choosing a product with a lower content of aluminium. Herein, we have significantly extended the scope of the previous research and the aluminium content of 30 of the most widely available and often used infant formulas has been measured.

**Methods:**

Both ready-to-drink milks and milk powders were subjected to microwave digestion in the presence of 15.8 M HNO_3_ and 30% *w/v* H_2_O_2_ and the aluminium content of the digests was measured by TH GFAAS.

**Results:**

Both ready-to-drink milks and milk powders were contaminated with aluminium. The concentration of aluminium across all milk products ranged from *ca* 100 to 430 μg/L. The concentration of aluminium in two soya-based milk products was 656 and 756 μg/L. The intake of aluminium from non-soya-based infant formulas varied from *ca* 100 to 300 μg per day. For soya-based milks it could be as high as 700 μg per day.

**Conclusions:**

All 30 infant formulas were contaminated with aluminium. There was no clear evidence that subsequent to the problem of aluminium being highlighted in a previous publication in this journal that contamination had been addressed and reduced. It is the opinion of the authors that regulatory and other non-voluntary methods are now required to reduce the aluminium content of infant formulas and thereby protect infants from chronic exposure to dietary aluminium.

## Background

In 2010 we published the aluminium content of 15 well known infant formula products [1]. We chose to identify the specific brands in order that consumers (more practically purchasers) of infant formulas might adopt a precautionary approach and choose those formulas with lower contents of aluminium. However, the range of values obtained was skewed towards high content and we were left to conclude that the aluminium content of infant formulas were too high, for example as compared to aluminium exposure through breast milk [2]. A recent report on Canadian infant formulas [3] has confirmed that this is likely to be a global as opposed to UK only problem. The interest in these data was overwhelming as evidenced by the paper being accessed via the journal website more than 20,000 times to-date [1] as well as myriad direct enquiries through email and other forms of communication. We should, perhaps, have not been surprised by the interest as according to a recent report by The Caroline Walker Trust [4], 25% of parents in the UK use formulas as the only source of ‘breast’ milk for infants from birth while 35% of parents use infant formulas from birth and more than 50% of infants of 4–10 weeks of age are fed solely on formulas. Given the high rate of use of infant formulas in the UK it was clear to us that a more comprehensive survey of the aluminium content of infant formulas was warranted. Herein we have reported the aluminium content of 30 infant formulas which are widely available in the UK.

## Methods

All products were bought off the shelf, stored according to manufacturers’ instructions and only opened at the time of sampling. The aluminium content of 10 ready-to-drink infant formulas and 20 powdered infant formulas were measured by transversely heated graphite furnace atomic absorption spectrometry (TH GFAAS) following acid/peroxide microwave digestion. All products were sampled directly from their containers following shaking to aid mixing of the products. For ready-to-drink products 1 mL 15.8 M HNO_3_ and 1 mL of 30% *w/v* H_2_O_2_ were added to 1 mL of milk and microwave assisted digestion carried out according to a previously validated method [5]. Digests were clear and made up to a total volume of 5 mL with ultrapure water (conductivity <0.067 μS/cm). For powdered milks an exact mass of powder, *ca* 0.5 g, was digested in the presence of 1 mL of 15.8 M HNO_3_ and 1 mL of 30% *w/v* H_2_O_2_ and the clear digest again made up to a total volume of 5 mL with ultrapure water. Total aluminium in 5 replicates of each product was measured by TH GFAAS using a previously validated and published method [5]. Quality assurance data for this method include an estimate for contamination, based upon 120 method blanks, of 54 ng per digest and this value was subtracted from each digest result to give final aluminium contents expressed either as μg/L (ready-to-drink) or μg/g (powders).

## Results

### Ready-to-drink infant formulas

The concentration of aluminium in ready-made infant formula milks ranged from 155 μg/L (Sma Toddler) to 422 μg/L (Aptamil Toddler Growing Up) (Table 1). The concentration data are arranged in ascending order and mean and SD are given for n = 5 replicates. The %RSD for all samples is consistently high and probably reflects the inhomogeneous distribution of aluminium throughout bulk volumes of product. Manufacturers’ recommendations for feeding were used along with the mean concentrations of aluminium to estimate daily exposures to aluminium from each product and these ranged from 86 (Hipp Organic First) to 127 (Cow & Gate First) μg Al/24 h at birth and 134 (Hipp Organic First) to 350 (Aptamil Follow-On) μg Al/24 h at 6 months of age (Table 1).

**Table 1:** The aluminium content of ready-made infant formula milks

**Commercial name of milk product (n = 5)**	**[Al] μg/L mean (SD)**	**Al μg/24 h**	
		**Birth**	**6 Mo.**
**Sma Toddler Milk**	155 (24)	NA	78^1^
**Hipp Organic First Infant Milk**	160 (67)	86	134
**Aptamil Hungry Milk**	163 (38)	88	171
**Sma First Infant Milk**	173 (34)	104	173
**Aptamil First Milk**	186 (37)	100	195
**Cow & Gate First Infant Milk**	235 (49)	127	226
**Sma Follow-On Milk**	271 (48)	NA	163
**Aptamil Follow-On Milk**	350 (100)	NA	350
**Cow & Gate Growing Up Milk**	380 (139)	NA	114^1^
**Aptamil Toddler Growing Up Milk**	422 (77)	NA	127^1^

^1^Data are for 12 months old. NA – not applicable.

### Powdered milk infant formulas

The concentration of aluminium in formulas made from powdered milks ranged from 0.69 μg/g (Hipp Organic Growing Up) to 5.27 μg/g (Cow & Gate Soya Infant Formula) (Table 2). The concentration data are arranged in ascending order and mean and SD are given for n = 5 replicates. The %RSD for all samples is consistently high and probably reflects the inhomogeneous distribution of aluminium throughout bulk volumes of the powders. Manufacturers’ recommendations for preparing milks were used to estimate the concentration of aluminium in the ready-to-drink product and these ranged from 118 (Aptamil Hungrier) to 756 (Cow & Gate Soya Infant Formula) μg/L at birth and 106 (Hipp Organic Growing Up) to 755 (Cow & Gate Soya Infant Formula) μg/L at 6 months of age. Manufacturers’ recommendations for feeding were used along with the mean concentrations of aluminium to estimate daily exposures to aluminium from each product and these ranged from 64 (Aptamil Hungrier) to 408 (Cow & Gate Soya Infant Formula) μg Al/24 h at birth and 80 (Hipp Organic Follow-On) to 725 (Cow & Gate Soya Infant Formula) μg Al/24 h at 6 months of age (Table 2).

**Table 2:** The aluminium content of powdered infant formula milks

**Commercial name of milk product (n = 5)**	**[Al] μg/g mean (SD)**	**[Al] in milk μg/L**		**Al μg/24 h**	
		**Birth**	**6 Mo.**	**Birth**	**6 Mo.**
**Hipp Organic Growing Up Milk**	0.69 (0.42)	NA	106^1^	NA	53^1^
**Aptamil Hungrier**	0.75 (0.25)	118	118	64	123
**Aptamil First Milk**	0.79 (0.46)	119	119	64	124
**Hipp Organic Follow-On**	0.85 (0.05)	NA	133	NA	80
**Sma First Infant**	0.86 (0.10)	120	120	65	116
**Sma Staydown**	0.86 (0.23)	123	123	67	118
**Aptamil (Colic & Constipation)**	1.01 (0.16)	155	155	84	149
**Cow & Gate for Hungrier Babies**	1.05 (0.09)	165	165	89	158
**Cow & Gate (Colic & Constipation)**	1.06 (0.48)	163	163	88	156
**Sma Extra Hungry**	1.13 (0.13)	158	158	86	152
**Cow & Gate Follow-On**	1.26 (0.15)	NA	206	NA	124
**Sma Follow-On**	1.28 (0.12)	NA	179	NA	108
**Aptamil Follow-On**	1.40 (0.33)	NA	229	NA	137
**Cow & Gate First Instant Milk**	1.44 (0.20)	216	216	117	207
**Hipp Organic Goodnight**	1.49 (0.54)	NA	229	NA	41
**Sma Lactose Free Formula**	1.98 (0.20)	284	284	153	273
**Sma Toddler**	2.74 (0.84)	NA	393	NA	212
**Hipp Organic First Infant**	2.80 (0.91)	411	411	222	345
**Sma Wysoy Soya Infant Formula**	3.92 (0.53)	656	654	354	627
**Cow & Gate Soya Infant Formula**	5.27 (0.96)	756	755	408	725

^1^Data are for 12 months old. NA – not applicable.

## Discussion

We have measured the aluminium content of the 30 most popular brands of infant formula available in the UK. We have included ready-to-drink products, which are available in both cardboard laminate cartons and plastic bottles, and milk powders, which are sold in tins (Sma), containers (Cow & Gate) and boxes (Hipp Organic). Generally the aluminium content of ready-to-drink products (Mean (SD) 250 (101) μg/L) were similar to the powdered milk products (Mean (SD) 246 (180) μg/L) though these ‘average’ values have no practical meaning as is clear from scrutiny of the data for the individual products (Tables 1 &2). The aluminium content of ready-to-drink milks was highest in the two products which were contained in plastic bottles (Cow & Gate Growing Up, Aptamil Toddler) as compared to long-life cartons. Both of the plastic bottles have a seal between the cap and the product which is made of aluminium foil and this may be a significant source of aluminium contamination in these milks. All of the long-life cartons are composed of classic trilaminate packaging which in each case includes an aluminium foil central layer. While this form of packaging is a source of aluminium contamination in the stored product it alone is unlikely to explain the wide difference in aluminium content of milks in similar packaging, for example, Sma Toddler milk and Aptamil Follow-On milk (Table 1).

The highest content of aluminium in powdered milks was found in both of the soya-based products (Table 2). It has been known for decades that soya is a significant source of aluminium contamination in infant formulas [6,7]. Powdered milk products are stored within 3 different containers and all of these include substantial amounts of aluminium in their packaging materials. Both Sma and Cow & Gate use containers which are lined with an aluminium-based composite and have a tear-away aluminium foil seal between the powder and the plastic lid. There are clearly opportunities for contamination of the stored milk powder by aluminium. Hipp Organic uses simple cardboard containers though the milk powders are actually contained in aluminium foil-based pouches inside these boxes. Again, this type of packaging may represent opportunities for contamination of the stored milk powder by aluminium [8]. While there have not been any scientific studies to determine if the packaging used for infant formula preparations is a source of aluminium contamination indirect confirmation of such was recently obtained by a reporter working for NBC in New York, USA where a formula manufacturer admitted that aluminium found in a customer’s powdered formula came from the aluminium-based packaging [9]. However, packaging is only one potential source of aluminium contamination in these milk powders as Hipp Organic supplies both the least (Hipp Organic Growing Up) and the most contaminated (Hipp Organic First Infant) of the non-soya-based milk powders and these products appear to use the same packaging materials. It should be of some concern that all of the infant formulas investigated herein are stored in containers which use a significant amount of aluminium-based packaging materials. The origins of the non-packaging-based contamination must include myriad ingredients used in these formulations and their processing to produce the final packaged product as those manufacturers that responded to our questions were adamant that aluminium in any form was not knowingly added to their formulas.

The concentration of aluminium in each of the 30 infant formulas is at least twice that which is recommended in the European Union for drinking water (50 μg/L) and in 14 of the milks it exceeds the maximum admissible level for drinking water of 200 μg/L [10]. While these recommended values for aluminium in drinking water were, historically at least, not set with human health as a criterion, they are used today in general practice to ascertain whether or not potable waters are fit for human consumption [10]. We consider that the aluminium content of all infant formulas investigated herein is too high and especially so considering that these products constitute either all or a substantial proportion of an infant’s diet in the first months of their lives. Based upon the criteria for drinking water they are not fit for human consumption and they contravene article 5 of the Food Safety Act which states that ‘infant formulas should not contain anything which might endanger the health of infants and young children’ [11]. Organisations such as the Food Standards Agency (FSA) in the UK and the EFSA in Europe have argued that the daily intake of aluminium from infant formulas is unlikely to exceed the tolerable weekly intake (TWI) of 1 mg/kg body weight set by the joint FAO/WHO Expert Committee on Food Additives [12]. However, these organisations, charged with the responsibility of protecting the public from additives in their food, need to recognise and emphasise that this value was determined for adults and, critically, it was not based upon any studies on humans, whether infants, adolescents or adults. The validity and usefulness of this TWI has repeatedly been questioned by those scientists working on human exposure to aluminium [13]. This issue can only be resolved through future research on infant exposure to aluminium through formula feeds and other routes. However, precautionary practical solutions to this public health issue should now be sought. The data published herein on the aluminium content of infant formulas can be used by parents and other interested parties as their best indicators of infant exposure to aluminium through formula feeds. Parents and others might now at the very least choose an appropriate formula with the lowest content of aluminium. Overall, the figures are slightly lower than the data we published in 2010 [1] and one would like to think that this is because manufacturers’ have taken precautions against the contamination of their products by aluminium. However, evidence of the widespread use of aluminium-based packaging does not support this. In addition, for these data we were able to apply a method blank approximation of aluminium contamination of the methods which was based upon 120 method blanks [5]. This value, which is based upon a much more rigorous statistical approach to levels of contamination in method blanks, was subtracted from the milk digests and was a greater value than that used for the previous data and so is probably a major factor behind the generally slightly lower aluminium contents reported herein. Clearly, the contamination of infant formulas by aluminium highlighted in this research can only be a snapshot of all formulas available at any one time. However, the consistency between the data in this study and our previous work [1] does suggest that the data are an accurate estimate of aluminium in infant formulas.

## Conclusions

We demonstrated previously that there was still too much aluminium in infant formulas. We elucidated the reasons why infant exposure to aluminium is an unnecessary potential health risk to children and may actually contribute towards ill health as adults. Herein we have re-visited this issue and we have found that many if not all infant formulas which are available in the UK are significantly contaminated with aluminium. All of these products are contained within aluminium-based packaging and this, alone, suggests that manufacturers’ have not yet addressed the problem of contamination of infant formulas by aluminium.

Given the lack of voluntary quality improvement by infant formula manufacturers, other public health initiatives, such as legislation/regulatory mandates and consumer advocacy and public awareness campaigns may be necessary to reduce the aluminium content of infant formulas to the lowest practical limit and thereby help to protect against chronic exposure of infants by aluminium.

## Competing interests

The authors have no competing interests.

## Authors’ contributions

CE designed the study, analysed the results and wrote the manuscript. NC, BP and BS collated the products, carried out the digestions and measured the Al content. All authors have read and approved the manuscript.

## Pre-publication history

The pre-publication history for this paper can be accessed here:

http://www.biomedcentral.com/1471-2431/13/162/prepub
